# Effect of the Coupling Agent (3-Aminopropyl) Triethoxysilane on the Structure and Fire Behavior of Solvent-Free One-Pot Synthesized Silica-Epoxy Nanocomposites

**DOI:** 10.3390/polym14183853

**Published:** 2022-09-15

**Authors:** Francesco Branda, Dambarudhar Parida, Robin Pauer, Massimo Durante, Sabyasachi Gaan, Giulio Malucelli, Aurelio Bifulco

**Affiliations:** 1 Department of Chemical Materials and Industrial Production Engineering (DICMaPI), University of Naples Federico II, Piazzale Vincenzo Tecchio 80, 80125 Naples, Italy; 2 Sustainable Polymer Technologies (SPOT) Team, Flemish Institute for Technological Research (Vito N.V.), Boeretang 200, 2400 Mol, Belgium; 3 Advanced Materials and Surfaces, Empa, Swiss Federal Laboratories for Materials Science and Technology, 8600 Dubendorf, Switzerland; 4 Laboratory for Advanced Fibers, Empa, Swiss Federal Laboratories for Materials Science and Technology, Lerchenfeldstrasse 5, 9014 St. Gallen, Switzerland; 5 Department of Applied Science and Technology, Politecnico di Torino, Viale Teresa Michel 5, 15121 Alessandria, Italy

**Keywords:** silica–epoxy nanocomposites, solvent-free one-pot process, sol-gel chemistry, nanoparticle formation mechanism, fire behavior

## Abstract

Uniformly distributed silica/epoxy nanocomposites (2 and 6 wt.% silica content) were obtained through a “solvent-free one-pot” process. The inorganic phases were obtained through “in situ” sol-gel chemistry from two precursors, tetraethyl orthosilicate (TEOS) and (3-aminopropyl)-triethoxysilane (APTES). APTES acts as a coupling agent. Surprisingly when changing TEOS/APTES molar ratio (from 2.32 to 1.25), two opposite trends of glass transformation temperature (Tg) were observed for silica loading, i.e., at lower content, a decreased Tg (for 2 wt.% silica) and at higher content an increased Tg (for 6 wt.% silica) was observed. High-Resolution Transmission Electron Microscopy (HRTEM) showed the formation of multi-sheet silica-based nanoparticles with decreasing size at a lower TEOS/APTES molar ratio. Based on a recently proposed mechanism, the experimental results can be explained by the formation of a co-continuous hybrid network due to reorganization of the epoxy matrix around two different “in situ” sol-gel derived silicatic phases, i.e., micelles formed mainly by APTES and multi-sheet silica nanoparticles. Moreover, the concentration of APTES affected the size distribution of the multi-sheet silica-based nanoparticles, leading to the formation of structures that became smaller at a higher content. Flammability and forced-combustion tests proved that the nanocomposites exhibited excellent fire retardancy.

## 1. Introduction

Hybrid organic/inorganic nanocomposite materials, with dispersed phases in sizes as small as a few nanometers, have recently raised great interest [1,2,3]. In this context, epoxy nanocomposites embedding different types of nanofillers represent a class of up-to-date materials with tailorable properties and wide applications in different sectors, including flame retardance [4,5], electromagnetic interference shielding [6], thermal and electrical conductivity [7], photonics [8], and energy storage [9], among others.

They are very advanced materials: their properties often go beyond the simple combination of each component [10]. A newly developed synthesis route exploits the sol-gel chemistry [11,12,13,14,15], where the mild synthesis conditions allow the “in situ” formation of the inorganic phase in the presence of a monomer or polymer system, through a “one pot” process [3,16]. Moreover, as long as the reaction rates are comparable, the simultaneous organic and inorganic synthesis gives rise to the formation of interpenetrating networks [16]. The sol-gel route allows the occurrence of the “bottom-up” approach [2,10], i.e., the building of the inorganic phase starting from simple low molecular-weight precursors. Epoxy–silica systems consisting of silica nanoparticles finely dispersed in a cured epoxy network are among the most studied organic–inorganic hybrid materials [4,5,6]. The sol-gel chemistry was largely exploited to produce them [4,15,17,18,19,20,21,22,23,24,25,26,27,28]. The inorganic phase is often obtained through hydrolysis and polycondensation reactions of alkoxysilanes. The reactions involving tetraethyl orthosilicate (TEOS) as the precursor are listed as follows [29,30,31,32,33,34]:

(1) $$\mathrm{Si}{\left(\mathrm{OR}\right)}_{4}+{\mathrm{nH}}_{2}O\;\to \;\mathrm{Si}{\left(\mathrm{OR}\right)}_{4-n}{\left(\mathrm{OH}\right)}_{n}+\mathrm{nROH}\;$$

(2) $$\equiv \mathrm{SiOH}+\mathrm{HO}-\mathrm{Si}\equiv \;\to \;\equiv \mathrm{Si}-O-\mathrm{Si}\equiv +{\;H}_{2}O\;$$

(3) ≡SiOH+RO−Si≡ → ≡Si−O−Si≡+ ROH 

Different strategies have been reported in the literature [4]. First, mixtures of TEOS and silane-functionalized epoxy were used to produce bi-continuous nanocomposites [18,19,20]. Then, epoxy/siloxane hybrids were produced in a two-step sol-gel process: the epoxy resin was added after hydrolysis and condensation of the inorganic precursors; the curing agent was added after removal of the hydro-alcoholic solvents [25,26]. Anti-corrosion hybrid coatings were prepared by adding a mixture of pre-hydrolyzed TEOS to silane functionalized epoxy [15,27,28].

Quite recently, the “solvent-free one-pot” process, which exploits the sol-gel technique, has been identified as a very promising approach for obtaining hybrid organic–inorganic systems [17,21,22,23,24]. In fact, this method allows for avoiding the use of high quantities of solvent, and therefore the removal, recycling, and disposal of this latter, which shows a significant environmental and economic impact. Further, the utilization of “solution blending” methods is quite complicated, as they require several processing steps for the synthesis of the nanoparticles, their functionalization, and the final removal of the solvent. These time-consuming steps are overcome in the “solvent-free one-pot” method, as the nanoparticle synthesis occurs in the presence of the resin and of a very limited amount of solvent (just required for the hydrolysis and condensation reactions) just before the curing process of the organic part of the nanocomposite. The “solvent-free one-pot” methodology has already been nicely reported in the literature. Matějka et al. [17] synthesized an organic–inorganic hybrid interpenetrating network by using TEOS as a precursor; both a one pot and a two-stage method were exploited. The investigation of the obtained networks revealed that the one-stage curing promoted the formation of large compact silica aggregates, 100–300 nm in diameter, while the two-stage process resulted in finer silica structures of 50–100 nm in diameter; therefore, the size of silica nanoparticles was significantly influenced by the synthesis methodology. Phonthamachai et al. [21] used TEOS in combination with (3-aminopropyl)trimethoxysilane (APTMS) to generate in situ oval-shaped silica nanoparticles in an epoxy resin. The silica–epoxy composite exhibited uniform dispersion and strong adhesion between the inorganic filler and epoxy matrix. Further, Afzal et al. [22] synthesized bi-continuous epoxy–silica hybrid polymers through functionalization of silica nanoparticles by (3-glycidoxypropyl)trimethoxysilane (GLYMO). It was found that the addition of GLYMO affected the particle size distribution, also increasing the glass transition temperature of entangled epoxy–silica networks.

In a related work, a “solvent-free one-pot” method was exploited for obtaining silica-epoxy HOI (silica content not exceeding 6 wt.% [35]. APTES (also behaving as a coupling agent) and TEOS were employed as precursors of the silica phase; their molar ratio was set at 1:2.32. No dripping phenomena were observed in vertical flame spread tests; moreover, as revealed by forced-combustion tests, the heat release rate (HRR) remarkably decreased (about −60%) as compared to the neat epoxy [35,36]. These findings were ascribed to the formation of a stable protective char. Moreover, in a further research effort [37], the concurrent presence of multi-sheet silica-based nanoparticles (as assessed by small- and wide-angle X-ray scattering and high-resolution transmission electron microscopy [38]) and P-based flame retardants highlighted the occurrence of synergism between the two components.

A mechanism was also proposed for the formation of multi-sheet silica nanoparticles in the solvent-free one-pot process [38]. On the basis of this research outcome, it was possible to prove the suitability of the proposed synthesis approach for the obtainment of other nanocomposite systems [39].

However, despite a lot of published literature on the development of silica nanocomposite via in situ sol-gel methodology, the effect of changing TEOS/APTES (coupling agent) ratio on the size and morphology of the silica nanoparticles is not well studied. In the present work, the effect of changing TEOS/APTES molar ratio (from 2.32 to 1.25), i.e., of the coupling agent (APTES) content, on the structure and fire behavior of silica epoxy nanocomposites produced through a solvent-free one-pot process is thoroughly investigated. The experimental results suggest that, as foreseen on the basis of the formation mechanism [38], the APTES content affects the size distribution of the multi-sheet silica-based nanoparticles, leading to the formation of structures that become finer when APTES loading increases, hence demonstrating that APTES plays an important role in the tuning of structure and properties of the final hybrid material. In fact, to the best of the authors’ knowledge, in the scientific literature, no examples concerning the control and design of nanoparticle distribution (number/size) are reported. Thus, the results of the present work may significantly help to better understand this challenging issue. This is of particular value when considering that the proposed synthesis strategy can be easily applied to other nanocomposites than silica/epoxy hybrids [39].

## 2. Materials and Methods

### 2.1. Materials

TEOS, >99%, APTES, >98%, and ethanol (ACS reagent, anhydrous) were purchased from Sigma-Aldrich (Switzerland). A commercially available epoxy system (SX10 by MATES S.r.l., Milan, Italy), made of bisphenol A diglycidyl ether (DGEBA) and modified cycloaliphatic polyamines, was employed for producing the epoxy network and its nanocomposites.

### 2.2. Synthesis and Preparation of Silica–Epoxy Nanocomposites

Table 1 collects the compositions of the reaction batches. Two series were produced by equimolarly replacing TEOS with APTES starting from a TEOS/APTES molar ratio equal to 2.32. The TEOS/APTES molar ratios investigated were: 1.25, 1.50, 1.75, 2.0, and 2.32. Decreasing values were chosen in the light of studying the effect of changing the ratio of precursor molecules in favor of the coupling agent (APTES). Therefore, the Si/EPO molar ratio was kept constant throughout each series. TEOS, distilled water, and ethanol (Table 1) were added to the silanized epoxy and stirred vigorously at 80 °C under reflux for 90 min. To remove ethanol and water, the reaction vessel was, then, opened and kept at 80 °C for 30 min. The amount of hardener needed for the curing was then added to the mixture at room temperature and mixed for 5 min. The resulting mixtures were degassed under vacuum and poured into a Teflon^®^ mold. The curing process was carried out at 30 °C/24 h and the samples were post-cured at 80 °C/4 h.

Considering a complete conversion of the precursors, the theoretical silica contents of the two series are 2 and 6%, respectively. The sample acronyms reported in the first column of Table 1 are coded based on the silica content and TEOS/APTES molar ratio: therefore, *EPO_X%Si_Y* indicates the HOI containing *X* wt.% of silica and with TEOS/APTES molar ratio equal to *Y*. The two series are indicated as *series a* (2 wt.% of silica) and *b* (6 wt.% of silica). The unmodified resin represents the cured pristine system, which will be indicated as “epoxy” and with the following acronym “EPO”.

### 2.3. Characterization and Investigation Techniques

A Nikolet 5700 FTIR spectrometer (Thermo Fisher, Waltham, MA, USA) working in ATR (Attenuated Total Reflectance mode, single reflection) was exploited for recording Fourier Transform Infrared (FTIR) spectra Resolution was set at 4 cm^−1^; 32 scans were performed and elaborated with Thermo Scientific™ OMNIC™ Software Suite (v7.2, Thermo Fisher, Waltham, MA, USA, 2005).

Dynamic Mechanical Analysis (DMA) was performed using a DMA3300 (TA Instruments, Eden Prairie, MN, USA) apparatus, working in three-point bending mode at 1 Hz frequency, from 25 to 100 °C at 3 K/min. The tests were repeated on three samples of each composition.

A TEM/STEM JEOL JEM 2200 fs microscope (Akishima, Tokyo, Japan) was employed for acquiring a High-Resolution Transmission Electron Microscopy (HRTEM) image of EPO_6%Si_2.32 sample (operating voltage: 200 kV). This latter was first ground and dispersed in water; then, one drop of the dispersion was deposited on a Lacey Carbon film copper TEM grid and dried overnight at 40 °C in an oven. The particle size and distribution of fifty particles randomly located were evaluated by means of Image J software; this software was also applied to a high-resolution image for evaluating the lattice plane distance.

To collect low-resolution TEM images, a bright field TEM microscopy (TEM, FEI Company, Hillsboro, OR, USA) analysis was performed on EPO _6%Si _2.32 with the usage of an FEI TECNAI G12 Spirit-Twin (LaB6 source) coupled with an FEI Eagle-4k CCD camera (FEI, Eindhoven, The Netherlands), setting the acceleration voltage to 120 kV.

The thermal and thermo-oxidative stability of the investigated materials was assessed by means of Thermogravimetric Analysis (TGA), with a Netzsch TG209 (Selb, Germany) apparatus; the samples were heated from room temperature to 800 °C, at 10 °C/min, under nitrogen or air atmosphere (experimental error: ±0.5 wt.% on mass, ±1 °C on temperature). The obtained data were elaborated with Proteus Software (v4.0, Labcenter Electronics Ltd., Yorkshire, UK, 2000).

The fire behavior of the synthesized systems was thoroughly assessed through both flammability (i.e., vertical flame spread tests, according to the IEC 60695-11-10 standard) and forced combustion tests (with a cone calorimeter, in accordance with the ISO 5660 standard). For the latter, a Noselab instrument (Nova Milanese, Italy) was utilized. The samples (50 × 50 × 2 mm^3^) were exposed to a 35 kW/m^2^ irradiative heat flux in a horizontal configuration. Time to ignition (TTI, s), the peak of heat release rate (pkHRR, kW m^−2^), time to peak (s), total heat release (THR, kW m^−2^), fire performance index (FPI, (kW m^−2^ s^−1^)), total smoke release (TSR, m^2^ m^−2^), specific extinction area (SEA, m^2^ kg^−1^), and the residues at the end of the tests were evaluated. Moreover, a pyrolysis combustion flow calorimeter (PCFC, Fire Testing Technology Instrument, London, UK) was employed to determine pkHRR, THR, and heat release capacity (HRC, J^−1^ g K), following the ASTM D7309 standard. Samples of about 7 mg were heated from 150 to 750 °C at 1 K/s in the pyrolysis zone. At least three tests were carried out on each material system and the results were averaged. In addition, the Flame Retardancy Index (FRI) was calculated [40,41] using the following equation:

(4) $$\mathrm{Flame}\;\mathrm{Retardancy}\;\mathrm{Index}\;=\frac{{\left[\mathrm{THR}\;\cdot \;\left(\frac{\mathrm{pHHR}}{\mathrm{TTI}}\right)\right]}_{\mathrm{Neat}\;\mathrm{Polymer}}}{{\left[\mathrm{THR}\;\cdot \;\left(\frac{\mathrm{pHHR}}{\mathrm{TTI}}\right)\right]}_{\mathrm{Composite}}}$$

## 3. Results and Discussion

### 3.1. Structure and Morphology of the Nanocomposites

**Figure 1:**
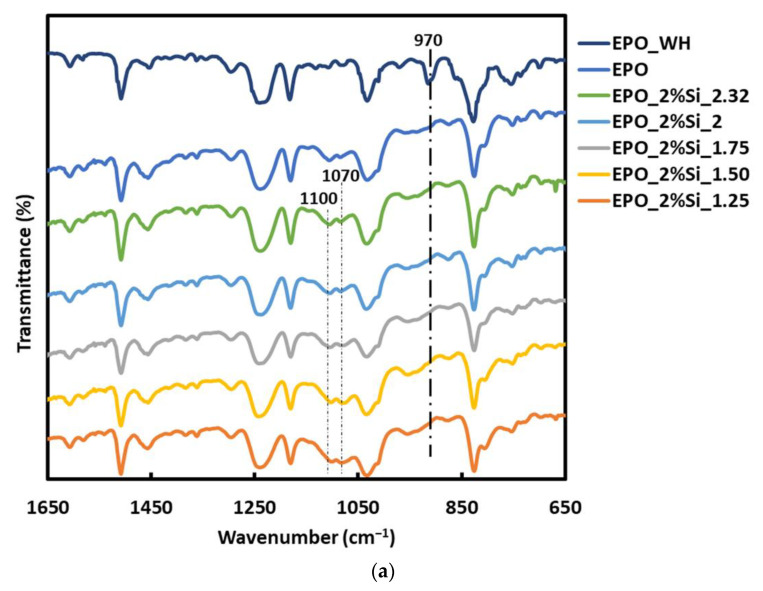

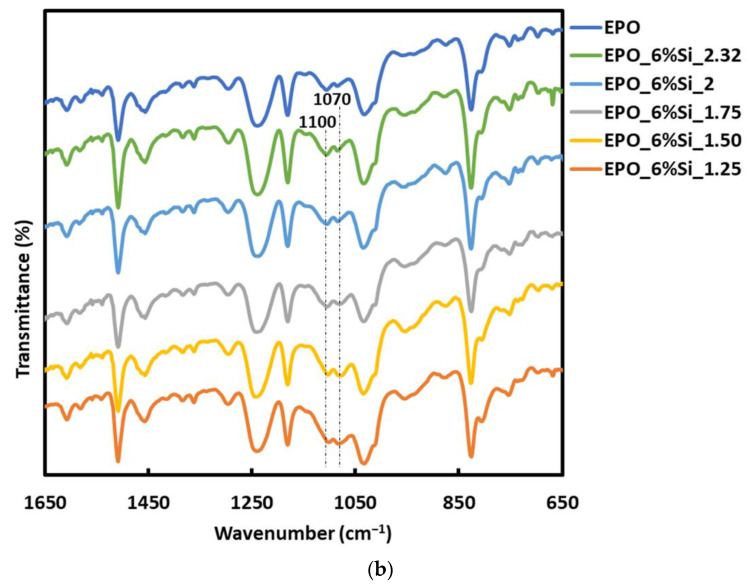
FTIR spectra of EPO_WH, EPO, and of the nanocomposites containing (**a**) 2 wt.% of silica and (**b**) 6 wt.% of silica, at different TEOS/APTS ratios.

The typical FTIR spectra of the nanocomposites containing 2 and 6 wt.% of silica are presented in Figure 1a,b, respectively, at different TEOS/APTES molar ratios. Moreover, the spectrum of the uncured resin (EPO_WH) is plotted in Figure 1a. The completeness of the curing reaction is clearly demonstrated by the disappearance of the band at 970 cm^−1^, ascribed to the epoxy group, in all the cured samples, in the adopted experimental conditions [24,42].

The figures also show that the bands at around 1070 and 1100 cm^−1^, attributed to SiO_4_ stretching vibration modes [43,44,45], progressively increase with increasing silica content. It is also worth reminding that the band attributed to silica shifts gradually toward lower wavenumbers and broadens upon the addition of alkaline and/or earth-alkaline oxides [43,44,45]. Indeed, the formation of SiO_4_ tetrahedral units bearing an increasing number of non-bridging oxygens determines a consequent progressive lack of network connectivity. This finding clearly supports the formation of silica derived from the two alkoxysilanes through the sol-gel reactions (Equations (1)–(3)). Moreover, by comparing samples at constant silica content, the band at 1070 cm^−1^ gradually increases with respect to that at 1100 cm^−1^, when TEOS/APTES ratio decreases. This finding can be attributed to the amino-propyl group of APTES, which is not hydrolyzable, hence decreasing the number of siloxane bridges per Si atom to three when silica is formed. This involves a loss of network connectivity during the equimolar substitution of TEOS with APTES, which justifies the appearance of the band at 1070 cm^−1^. However, no progressive wavenumber shift is observed when the TEOS/APTES ratio changes. Conversely, the band at 1070 cm^−1^ progressively increases with respect to that at 1100 cm^−1^, thus suggesting that two silicatic phases are concurrently formed; one of them mainly derives from APTES. This is in good agreement with recent results. In fact, HRTEM and SAXS/WAXS measurements proved that the solvent-free one-pot process leads to the formation of two silicatic phases, namely micelles mainly constituted of APTES and multi-sheet silica-based nanoparticles [38].

**Figure 2:**
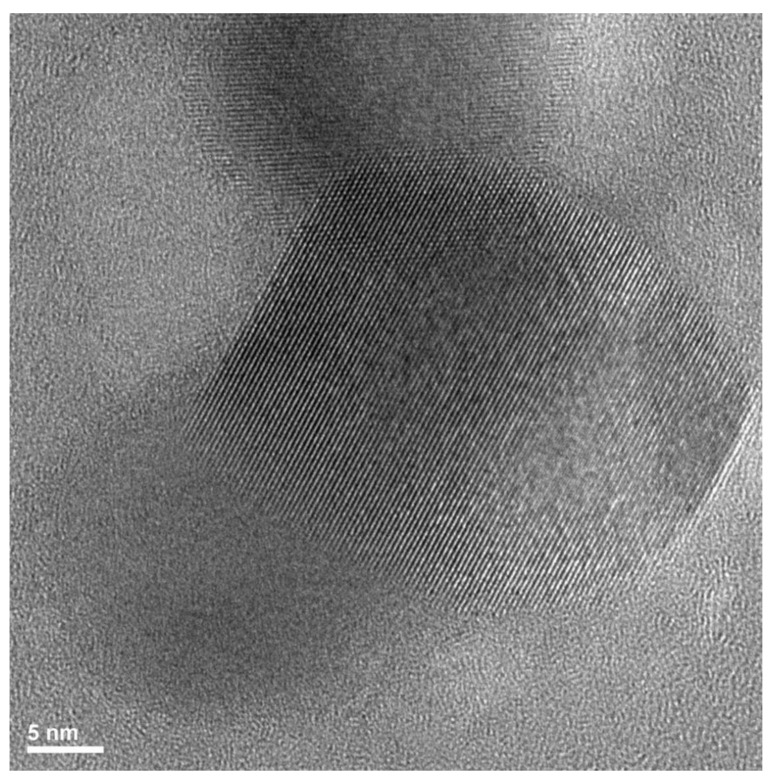
HRTEM micrograph of EPO_6%Si_2.32.

To study the effect of TEOS/APTES molar ratio on the multi-sheet silica-based nanoparticles, the EPO_6%Si_2.32 sample was investigated by TEM and HRTEM. The results were compared to those for EPO_6%Si_1.25, reported in a previous paper [38]. The TEM images at lower magnifications reported in Figure S1 show a very fine distribution of nanoparticles in the polymer matrix, with a limited number of clusters. Figure 2 shows a typical HRTEM micrograph of EPO_6%Si_2.32.

It shows a morphology consisting of multi-sheet nanoparticles, with a sheet thickness (determined according to the method developed by Albers [46] of about 0.33 nm, close to that measured, in the same way, for EPO_6%Si_1.25 [38]. However, the nanoparticle shown in Figure 2, which is representative of all the collected ones for EPO_6%Si_2.32, is about 30 nm, i.e., much larger with respect to those reported for 1.25 TEOS/APTES molar ratio (9 nm) [38].

### 3.2. Dynamic-Mechanical Behavior

**Figure 3:**
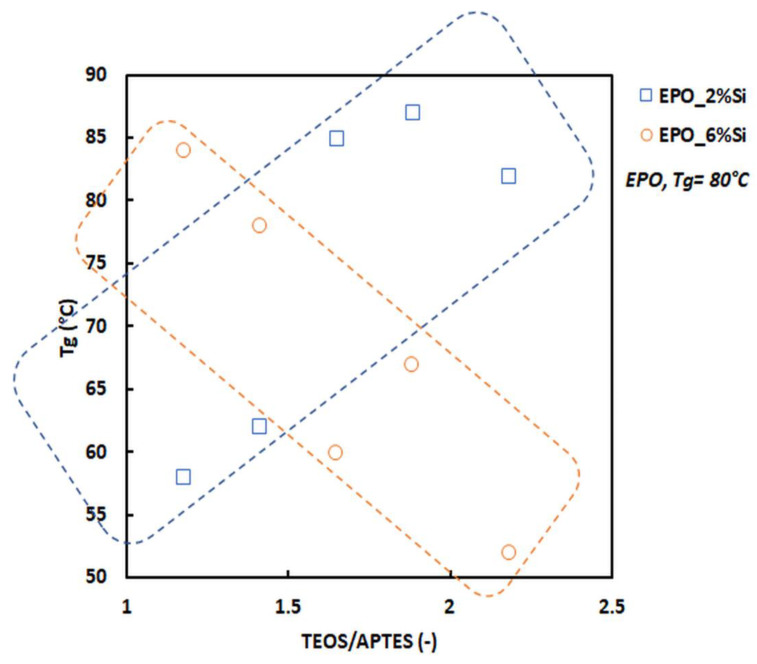
T_g_ vs. TEOS/APTES molar ratio for the nanocomposites at 2 (blue labels) and 6 wt.% (orange labels) of silica. In particular, blue squares represent the samples containing 2 wt.% of silica; orange circles indicate samples containing 6 wt.% of silica. The dotted blue line highlights the T_g_ values of all formulations containing 2 wt.% of silica at different TEOS/APTES ratios, while the dotted orange line identifies the T_g_ values of all formulations containing 6 wt.% silica at different TEOS/APTES ratios.

Dynamic-mechanical analyses were performed to evaluate the glass transition temperatures of the hybrid materials. Tanδ vs. temperature curves are presented in Figure S2. Figure 3 shows the T_g_ values (calculated as the maximum of the Tanδ peak) as a function of the TEOS/APTES molar ratio for the two prepared series.

The observed strong effects of the presence of coupling agents on T_g_ are consistent with literature data. In particular, the modification of epoxy resin by APTES generates APTES-modified epoxy resin improving the interphase and thus the adhesion between viscose fiber and polymer matrix in warp knitted viscose fabric composites [47]. The use of APTES can influence the chemistry of nanocomposites also through the formation of tailored phases in fiber-reinforced epoxy composites. As an example, jute fibers were surface-modified with APTES to produce polylactic acid-based composites showing boosted mechanical properties, thermal stability, and a strong interface between the components [48]. Therefore, APTES can be successfully exploited as a coupling agent for obtaining functional nanofillers, homogeneously dispersed in different polymer systems.

It is worth underlining that the sharp Tanδ peak of the cured epoxy (Figure S2 in the Supplementary Materials) becomes broader in the nanocomposites, with the appearance of some shoulders. This behavior, already reported in the literature as far as epoxy-polyurethane interpenetrating polymer systems are concerned [49], can be attributed to the formation of a co-continuous structure made of two different phases: one is made of more flexible segments, while the other contains more rigid structures.

In the present work, the formation of this co-continuous hybrid network can be attributed to the reorganization of the epoxy matrix around the two different silicatic phases identified by means of FTIR spectroscopy; this reorganization may be expected to be different and characterized by a different internal cohesion. Figure 3 highlights two opposite trends of T_g_ when the silica loading changes from 2 to 6 wt.%: in fact, the higher the TEOS/APTES molar ratio, the higher the T_g_ for *series a* (2 wt.% silica), and the lower for *series b* (6 wt.% silica). In other words, at 2 wt.% silica, increasing the APTES content promotes the decrease in T_g_: this finding is consistent with the FTIR results that evidenced an increase in the looser silica content when APTES content increases. Moreover, the HRTEM observations and the T_g_ changes for the 6% silica content may be explained on the basis of the size of the nanoparticles that become smaller, but their number increases when APTES content increases. In fact, a finer microstructure (more particles, but with a smaller size), leads to a greater interface per unit volume and, therefore, to a T_g_ increase. These results are clearly in good agreement with a mechanism recently proposed [38] that is shortly reminded in the following. At the beginning of the solvent-free one-pot process, a hybrid epoxy-APTES molecule is formed. Upon addition of TEOS, water, and alcohol, the hybrid molecule acts as a surfactant, allowing the formation of micelles in the epoxy matrix. The micelles would collect the added water and ethanol, thus giving rise to the nano-environment where TEOS, originally located in the epoxy, can undergo full hydrolysis and then poly-condensation, giving rise to the formation of silica-based oligomers according to Equations (1)–(3).

**Figure 4:**
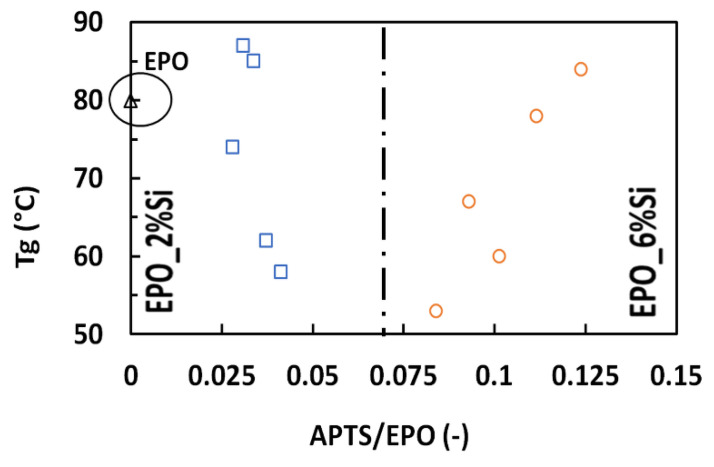
T_g_ vs. APTES/epoxide weight ratio. In particular, blue squares represent samples containing 2 wt.% silica; orange circles indicate samples containing 6 wt.% silica and the black triangle displays pristine resin.

In order to successfully explain crystallization processes, Tamman and other researchers [50,51,52,53] have proposed a mechanism of nucleation and successive growth, which further supports the morphology of the structures shown in Figure 2. In particular, in the present system, nucleation would involve the aggregation of micelles until the achievement of a critical size allowing the formation of the nuclei of the phases observed in HRTEM [38]. When the critical size is reached, the micelle aggregates will disaggregate into nanoparticles (i.e., the nuclei of new phases) and smaller micelles containing the water residual from the hydrolysis and polycondensation reactions. Conversely, nanoparticle growth would occur through the addition of “smaller structural units” present in the matrix to the already formed nanoparticle surface. The “smaller structural units” would be the fully hydrolyzed silica-based oligomers formed in the micelles [38]. Upon aggregation of the micelles with the already formed nanoparticles, the structural units therein present would be transferred, thus allowing nanoparticle growth. Based on this mechanism, the system would finally consist of both micelles and nanoparticles distributed in the epoxy network. The micelles are mainly derived from APTES and, therefore, should correspond to the looser silicatic phase hypothesized above. Unlike nanoparticles, their presence is expected to lower the T_g_. An important parameter refers to the APTES/Epoxy weight ratio. In fact, increasing the APTES content supports the increase in the micelle concentration that favors both nucleation and growth rates of nanoparticles to increase. If the mechanism is correct, the dependence of the nanoparticle growth rate is expected to be first order on micelle concentration, while that of the nucleation rate is expected higher. Therefore, increasing micelle concentration accounts for higher growth of the nucleation rate with respect to crystal growth. Thus, the crystal size distribution may be expected to become finer when APTES content increases. The whole T_g_ results are better interpreted when they are plotted as a function of the APTES/Epoxide weight ratio (Figure 4).

When the concentration of micelles is very low, the negative effect of micelle concentration on T_g_ is predominant: this occurs for the samples of *series a*. The increase in micelle concentration accounts for the increase in the nucleation and crystal growth, making prevalent the effect of nanoparticle size distribution: this occurs for samples of *series b*. As a consequence, the plot of Figure 4 shows a minimum that corresponds to an APTES/epoxide weight ratio of 0.06.

### 3.3. Thermal and Fire Behavior

Table S1 collects the results from thermogravimetric analyses for some of the investigated samples. The thermal analysis was carried out on EPO, EPO_2%Si_2.32, and EPO_6%Si_2.32, which were selected as representative of all the other formulations. The thermal behavior of pristine resin and silica–epoxy nanocomposites can be explained on the basis of the degradation mechanism reported in the literature [54]. In an inert atmosphere, the EPO and its nanohybrids decompose through a single step at about 350 °C, which is in agreement with the degradation of epoxy resins cured with aliphatic hardeners [55]. The generation of in situ silica in the epoxy matrix confers acidic characteristics to the hybrids, which anticipates the carbonization process via dehydration of epoxy resin, resulting in a lower value of T_5%_ for both EPO_2%Si_2.32 and EPO_6%Si_2.32, compared to the neat epoxy network. This effect is also observed in air. With respect to EPO, a significant increase in the residues at 800 °C, in both atmospheres, was observed for the silica-epoxy nanocomposites, which can be ascribed to the silica phase exerting a thermal shield effect on the char during the heating up [56]. The presence of oxygen reduces the formation of a stable char and lowers the values of the residual masses, as all the aromatic moieties undergo a full oxidation process at high temperatures [57]. Finally, the in situ modification of epoxy chains improves the overall thermal stability due to the silica phase acting as a barrier, slowing down the diffusion of volatiles and oxygen from air into the polymer bulk and vice versa. This role of silica is mainly observed in the case of EPO_6%Si_2.32, because of its high silica content.

The flame-retardant properties provided to the epoxy network by the “in situ” formation of the silica phases have been thoroughly investigated by means of both flammability and forced-combustion tests.

The results of vertical flame spread tests, collected in Table 2, show a slight improvement in the UL94 rating only for the hybrids containing 6 wt.%. of silica at lower TEOS/APTES molar ratios (i.e., 1.25 and 1.75), which achieve no-dripping V-1 rating. More in detail, EPO_6%Si_1.25 and EPO_6%Si_1.75 do not completely burn up to the holding clamp; therefore, the upper part of the specimens still appears as before the test. In addition, the samples do not drip during the combustion, and no ignition of cotton batting occurs. Further, the samples containing 2 wt.% of silica do not exhibit melt dripping phenomena, as well as those containing 6 wt.% of silica, and with a TEOS/APTES molar ratio of 2.32, though the latter cannot be rated.

**Figure 5:**
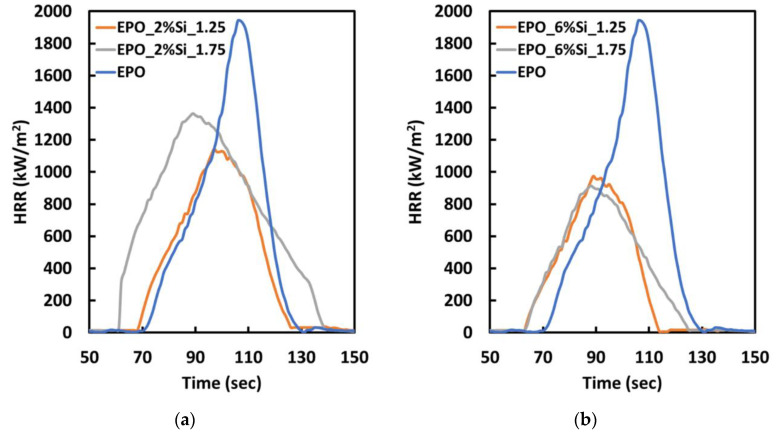
Typical HRR curves vs. time for EPO and some nanocomposites containing (**a**) 2 wt.% of silica and (**b**) 6 wt.% of silica, at different TEOS/APTS ratios.

Cone calorimetry tests under 35 kW/m^2^ irradiative heat flux were performed to gain more insight into the combustion behavior of the composites. The results are summarized in Table 3 and Table 4. Figure 5 displays some typical HRR vs. time curves. First of all, it is worthy to note that all the nanocomposites accelerate the ignition with respect to the neat epoxy resin: this is attributable to the acidic character of the “in situ” formed silica phases, which catalyzes the pyrolysis of the epoxy network [35,58,59,60,61]: this effect is more pronounced when TEOS/APTES molar ratio increases, as the APTES precursor, which is less acidic than TEOS, is replaced in the precursor mixture.

The values of FRI are within 1 and 10, indicating that all the investigated nanocomposites can be classified as “good”. Finally, the formation of the silica phases, regardless of the sol-gel recipe, does not seem to affect the smoke parameters (Table 4), which do not show a clear trend, being almost unchanged.

**Figure 6:**
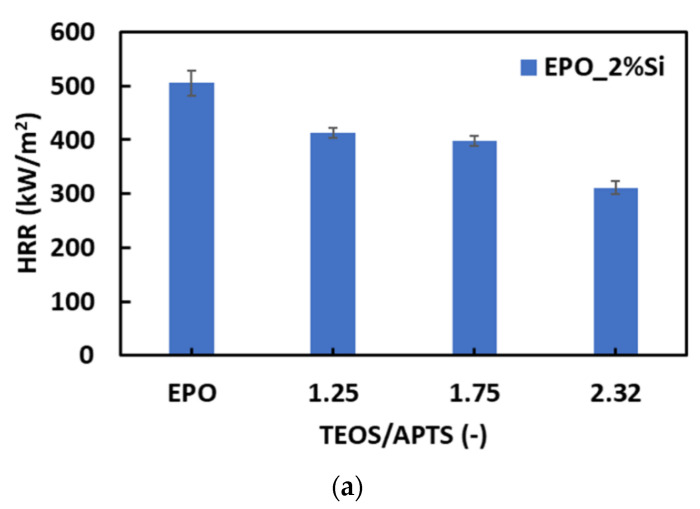

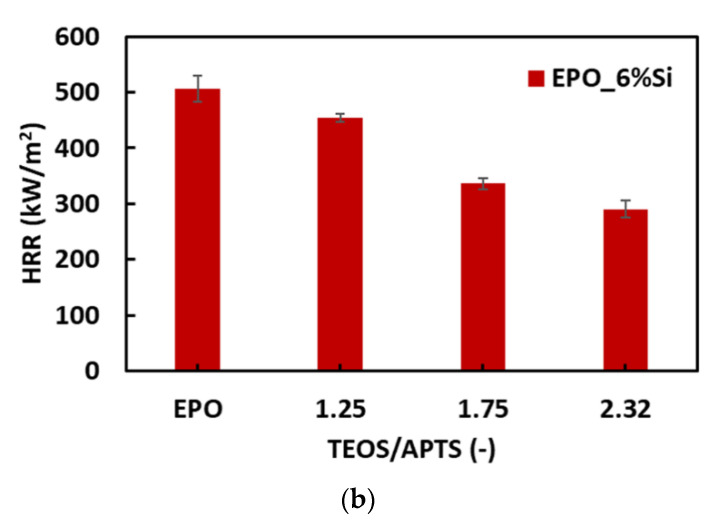
HRR vs. TEOS/APTES molar ratio for the two investigated series containing (**a**) 2 wt.% of silica and (**b**) 6 wt.% of silica.

It is worth noting the effect of the TEOS/APTES molar ratio on the HRR values: as depicted in Figure 6, HRR decreases with increasing TEOS/APTES molar ratio, irrespective of the silica content. Similar results were observed in the case of pkHRR parameter (Table 3 and Figure S3 in the Supplementary material), which strongly decreases when silica–epoxy composites are considered, especially for systems with the highest silica content (6 wt.%). Samples with the highest TEOS/APTES molar ratio (EPO_2%Si_2.32 and EPO_6%Si_2.32) also show very low THR values compared to the pristine resin and other silica–epoxy composites, probably due to the flame-retardant action of silica nanoparticles on the underlying polymer matrix (Figure S3). The presence of the silica phase guarantees lower heat exchange during the combustion [62]. The acidic characteristic of the in situ generated sol-gel silica phase affects the TTI values of the silica–epoxy nanocomposites, which appear notably reduced with respect to EPO. These results are in agreement with thermogravimetric measurements, as the presence of silica nanoparticles accelerates the degradation of the polymer matrix and thus its ignition (Table S1 and Figure S3).

**Figure 7:**
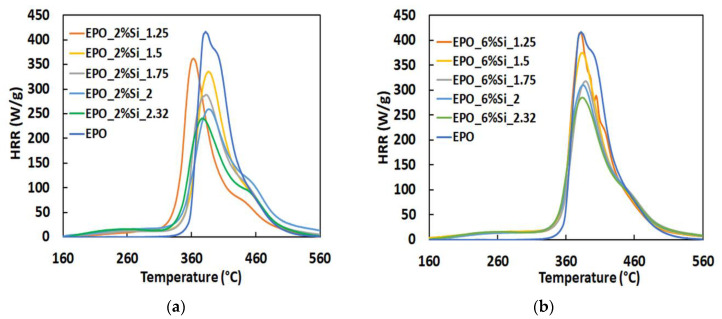
Typical HRR curves vs. temperature for EPO and some nanocomposites containing (**a**) 2 wt.% of silica and (**b**) 6 wt.% of silica, at different TEOS/APTS ratios.

Table 5 and Figure 7 summarize the results obtained from Pyrolysis Combustion Flow Calorimetry tests. The PCFC instrument permits to obtain different information on the fire behavior of composites, with respect to cone calorimeter tests. The cone calorimeter simulates a real fire event, as the combustion of the tested sample occurs continuously and in the presence of air through the use of a heat radiation source and a spark ignition for the flame. Conversely, PCFC equipment allows for a non-flaming test, where the condensed phase and gas phase processes of flaming combustion are separately reproduced. PCFC measurements involve a controlled pyrolysis of the sample in an inert gas stream followed by high-temperature oxidation of the volatile pyrolysis products [63]. Therefore, the PCFC can be very useful in investigating the gas phase action of flame retarded systems as compared with cone calorimetry.

Despite the huge differences between PCFC and cone calorimetry analyses [64], the results of the former further confirm the flame retardant effect provided by the silica phases in the condensed phase, as witnessed by the decrease in both HRC and pkHRR values of the nanocomposites with respect to neat EPO. In particular, both the HRC and the pkHRR, being constant the silica content, substantially monotonically decrease with increasing the TEOS/APTES molar ratio. The protection exerted by the silica phases is also evidenced by the significant increase in the residues at the end of PCFC and thermogravimetric analyses. These results confirm the flame-retardant mechanism exerted by silica nanoparticles during the combustion process. The ceramic layer of silica acts as a barrier for oxygen and heat during the degradation, which results in a remarkable decrease in HRC values, especially at high TEOS/APTES ratios for both series of samples.

## 4. Conclusions

Silica/epoxy nanocomposites (silica content ranging from 2 to 6 wt.%) were successfully obtained through an in situ sol-gel “solvent-free one-pot” process, using TEOS and APTES as precursors in a molar ratio changing from 1.25 to 2.32. According to a recently proposed mechanism of nanoparticles formation, the obtained results suggest the formation of a co-continuous hybrid network attributable to the reorganization of the epoxy matrix around two different silicatic phases, namely micelles formed mainly by APTES and multi-sheet silica nanoparticles. The coupling agent plays an important role in tuning the structure and properties of the prepared hybrid materials, hence making it possible to control and design the nanoparticles distribution (number/size). The main outcomes can be summarized as follows:

- The APTES content affects the size distribution of the multi-sheet silica-based nanoparticles, leading to the formation of finer structures (more particles but with smaller sizes) by increasing the APTES loading.
- Despite the very low silica content, the nanocomposites exhibit an excellent flame retardance, that, depending on the composition, in the vertical frame tests, is witnessed by the absence of dripping and, in some cases, by V1 rating.
- As assessed by cone calorimetry tests, a significant decrease in the heat release rate (up to 60%) with respect to the neat epoxy network is observed. The HRR is strongly affected by TEOS/APTES molar ratio.
- The flame retardancy index values are within 1 and 10, thus indicating that all the investigated nanocomposites can be classified as “good”.

Due to the flame-retardant mechanism of silica nanoparticles exerted in the condensed phase, the sample with the highest content of silica (6 wt.%) shows the best overall performance in terms of fire retardance.

## Figures and Tables

**Table 1:** Composition and codes of the investigated epoxy samples.

Sample	Epoxy (g)	Hardner (g)	APTS (g)	TEOS (g)	APTS/EPO (-)	EtOH (g)	Water (g)
**EPO**	15	3.9	-	-	0.000	-	-
***Series a***							
**EPO_2%Si_1.25**	15	3.9	0.619	0.728	0.041	0.081	0.403
**EPO_2%Si_1.50**	15	3.9	0.557	0.786	0.037	0.087	0.408
**EPO_2%Si_1.75**	15	3.9	0.506	0.834	0.034	0.092	0.412
**EPO_2%Si_2.00**	15	3.9	0.464	0.874	0.031	0.097	0.415
**EPO_2%Si_2.32**	15	3.9	0.420	0.916	0.028	0.101	0.419
***Series b***							
**EPO_6%Si_1.25**	15	3.9	1.857	2.185	0.124	0.242	1.208
**EPO_6%Si_1.50**	15	3.9	1.671	2.359	0.111	0.261	1.223
**EPO_6%Si_1.75**	15	3.9	1.519	2.502	0.101	0.277	1.235
**EPO_6%Si_2.00**	15	3.9	1.393	2.621	0.093	0.290	1.246
**EPO_6%Si_2.32**	15	3.9	1.259	2.748	0.084	0.304	1.257

**Table 2:** Results of vertical flame spread tests for EPO and some nanocomposites.

Sample	UL 94/Dripping
EPO	*Not classifiable (NC)/Yes*
EPO_2%Si_1.25	NC/No
EPO_2%Si_1.75	NC/No
EPO_2%Si_2.32	NC/No
EPO_6%Si_1.25	V1/No
EPO_6%Si_1.75	V1/No
EPO_6%Si_2.32	NC/No

**Table 3:** Results (thermal parameters) from cone calorimetry tests (irradiative heat flux: 35 kW/m^2^).

Sample	TTI (s)	HRR (kW/m^2^)	pkHRR (kW/m^2^)	THR (MJ/m^2^)	Residue (wt.%)	FRI (-)
**EPO**	70 ± 3.2	506 ± 23.3	1941 ± 384	83 ± 3.1	3 ± 0.7	-
**EPO_2%Si_1.25**	68 ± 5.1	413 ± 10.2	1114 ± 108	92 ± 6.9	7 ± 0.7	2.1
**EPO_2%Si_1.75**	61 ± 4.5	398 ± 9.3	1353 ± 98	106 ± 5.7	7 ± 0.6	1.7
**EPO_2%Si_2.32**	37 ± 4.1	311 ± 12.1	991 ± 73	67 ± 9.2	6 ± 0.5	2.3
**EPO_6%Si_1.25**	63 ± 7.3	454 ± 7.6	923.3 ± 106	107 ± 8.2	10 ± 0.6	2.4
**EPO_6%Si_1.75**	59 ± 8.1	336 ± 10.2	902.3 ± 107	98 ± 6.9	9 ± 0.7	2.5
**EPO_6%Si_2.32**	32 ± 2.1	290 ± 15.2	1231 ± 228	57 ± 6.2	10 ± 0.6	1.9

**Table 4:** Results (smoke parameters) from cone calorimetry tests (irradiative heat flux: 35 kW/m^2^).

Sample	TSR (m^2^/m^2^)	SEA (m^2^/kg)	CO Yield (kg/kg)	CO_2_ Yield (kg/kg)	CO/CO_2_ (-)
**EPO**	3066 ± 206	940 ± 36	0.061 ± 0.03	2.08 ± 0.06	0.029
**EPO_2%Si_1.25**	3306 ± 223	887 ± 11	0.059 ± 0.02	2.07 ± 0.04	0.028
**EPO_2%Si_1.75**	3851 ± 157	875 ± 15	0.053 ± 0.01	1.97 ± 0.03	0.026
**EPO_2%Si_2.32**	2604 ± 291	941 ± 38	0.060 ± 0.04	1.94 ± 0.03	0.031
**EPO_6%Si_1.25**	4053 ± 178	932 ± 18	0.054 ± 0.02	1.93 ± 0.02	0.027
**EPO_6%Si_1.75**	4310 ± 125	967 ± 10	0.047 ± 0.03	1.89 ± 0.04	0.024
**EPO_6%Si_2.32**	2087 ± 355	895 ± 31	0.063 ± 0.01	1.95 ± 0.05	0.032

**Table 5:** PCFC data for epoxy samples containing 2 and 6 wt.% of silica.

Sample	THR (kJ/g)	HRC (J/g-K)	pkHRR (W/g)	Residue (wt.%)
**EPO**	26.7 ± 0.61	427 ± 12.2	431 ± 12.2	5.05 ± 0.87
**EPO_2%Si_1.25**	26.3 ± 0.52	424 ± 1.80	364 ± 0.54	12.6 ± 1.39
**EPO_2%Si_1.50**	25.7 ± 0.78	381 ± 7.76	336 ± 7.31	14.2 ± 2.27
**EPO_2%Si_1.75**	25.1 ± 0.36	336 ± 5.15	298 ± 6.66	16.3 ± 0.17
**EPO_2%Si_2.00**	24.7 ± 0.39	299 ± 3.40	263 ± 4.89	16.4 ± 0.38
**EPO_2%Si_2.32**	24.3 ± 0.12	284 ± 11.4	255 ± 9.28	17.3 ± 0.91
**EPO_6%Si_1.25**	28.9 ± 0.21	460 ± 14.8	417 ± 14.6	9.66 ±0.77
**EPO_6%Si_1.50**	27.1 ± 0.62	436 ± 4.03	373 ± 3.13	11.1 ± 2.04
**EPO_6%Si_1.75**	25.1 ± 0.33	345 ± 15.2	304 ± 11.4	14.8 ± 0.46
**EPO_6%Si_2.00**	24.8 ± 0.57	324 ± 26.5	283 ± 19.8	14.6 ± 0.66
**EPO_6%Si_2.32**	25.1 ± 0.16	332 ± 4.64	291 ± 4.94	13.9 ± 0.48

## Data Availability

Not applicable.

## Supplementary Materials

The following supporting information can be downloaded at: https://www.mdpi.com/article/10.3390/polym14183853/s1, Figure S1: Low-resolution TEM micrographs of EPO _6%Si _2.32 at different magnifications.; Figure S2: Tanδ vs. temperature curves of the investigated samples; Table S1: TGA analysis of all samples (TEOS/APTS = 2.32) in air and N_2_. T_5%,_ T_10%_ and T_50%_ are the temperatures at which 5 wt.%, 10 wt.% and 50 wt.% losses were recorded, respectively. T_peak_ is the temperature, at which the weight loss rate reached a maximum; the residues at T_peak_ and 800 °C are also reported. Figure S3: THR vs. TEOS/APTES molar ratio for the two investigated series containing 2 wt.% of silica and 6 wt.% of silica. TTI vs. TEOS/APTES molar ratio for the two investigated series containing 2 wt.% of silica and 6 wt.% of silica. pkHRR vs. TEOS/APTES molar ratio for the two investigated series containing 2 wt.% of silica and 6 wt.% of silica.
