# Supplementary material for: Effect of the Coupling Agent (3-Aminopropyl) Triethoxysilane on the Structure and Fire Behavior of Solvent-Free One-Pot Synthesized Silica-Epoxy Nanocomposites
Source: Polymers (Basel). 2022 Sep 15;14(18):3853. doi: 10.3390/polym14183853 (PMC9506363; doi:10.3390/polym14183853)
Supplement: Supplementary file 1 [file polymers-14-03853-s001.zip › polymers-1875198-supplementary.pdf]

Supplementary material

Effect of the Coupling Agent (3-aminopropyl) triethoxysilane on the Structure and Fire Behavior of Solvent-Free One-Pot Synthesized Silica-Epoxy Nanocomposites

Francesco Branda [1,*], Dambarudhar Parida [2], Robin Pauer [3], Massimo Durante [1], Sabyasachi Gaan [4], Giulio Malucelli [5], Aurelio Bifulco [1,*]

[1] Department of Chemical Materials and Industrial Production Engineering (DICMaPI), University of Naples Federico II, Piazzale Vincenzo Tecchio 80, 80125 Fuorigrotta, Naples, Italy
[2] Sustainable Polymer Technologies (SPOT) Team, Flemish Institute for Technological Research (Vito N.V.), Boeretang 200, 2400 Mol, Belgium
[3] Advanced Materials and Surfaces, Empa, Swiss Federal Laboratories for Materials Science and Technology, CH-8600 Dubendorf, Switzerland
[4] Laboratory for Advanced Fibers, Empa, Swiss Federal Laboratories for Materials Science and Technology, Lerchenfeldstrasse 5, 9014 St. Gallen, Switzerland
[5] Department of Applied Science and Technology, Politecnico di Torino, Viale Teresa Michel 5, 15121 Alessandria, Italy
* Correspondence: branda@unina.it (F.B.); aurelio.bifulco@unina.it (A.B.); Tel.: +39-081-768-2412 (F.B.); +39-081-768-2413 (A.B.)

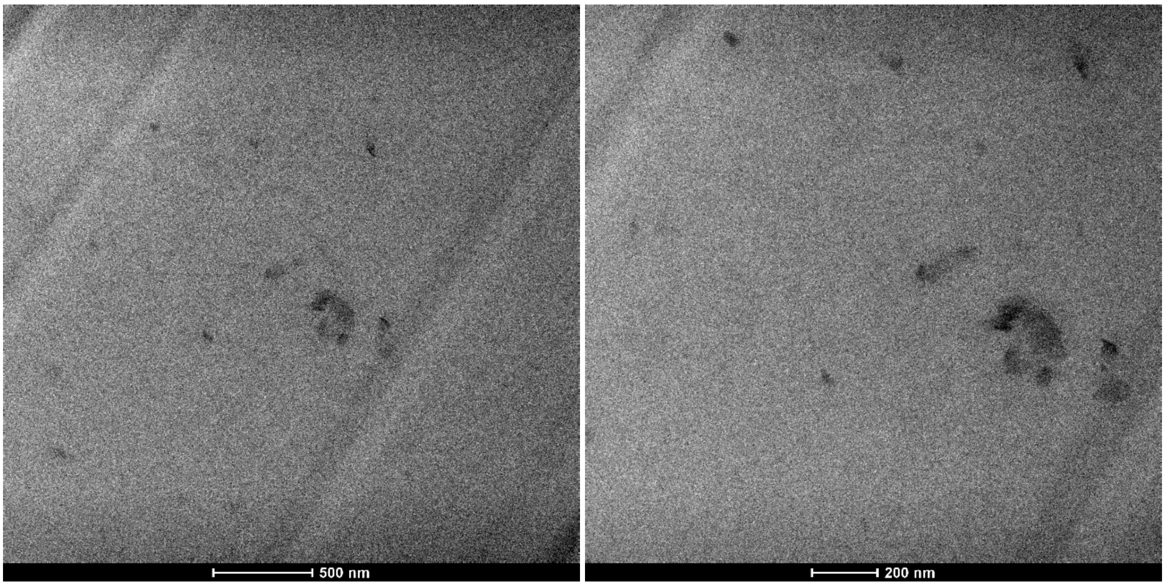

(a) (b)

Figure S1. Low-resolution TEM micrographs of EPO _6%Si _2.32 at different magnifications.

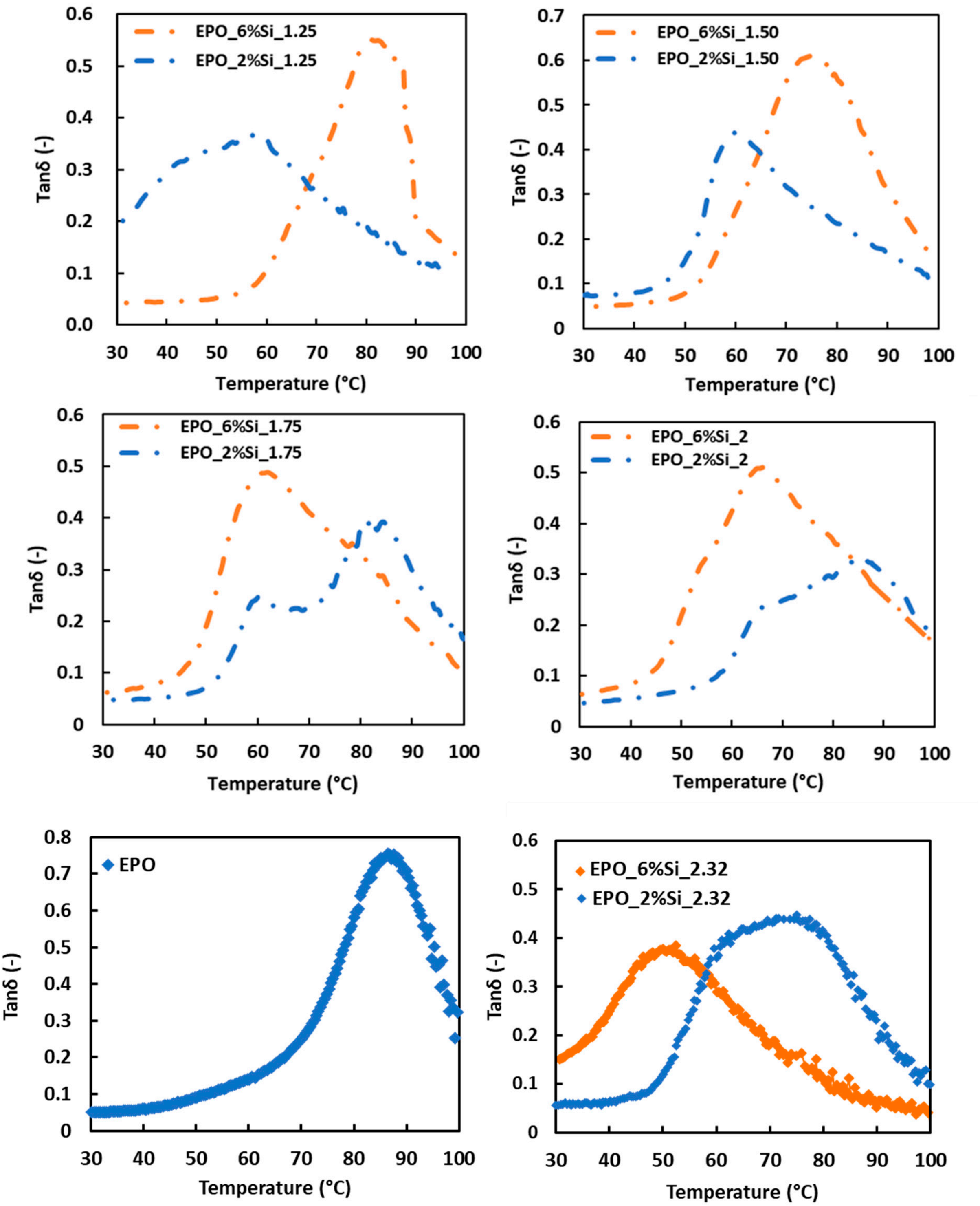

Figure S2. Tanδ vs. temperature curves of the investigated samples.

Table S1. TGA analysis of all samples (TEOS/APTS=2.32) in air and N_2. $T_{5\%}$, $T_{10\%}$ and $T_{50\%}$ are the temperatures at which 5 wt.%, 10 wt.% and 50 wt.% losses were recorded, respectively. T_{peak} is the temperature, at which the weight loss rate reached a maximum; the residues at T_{peak} and 800 °C are also reported.

Sample	$T_{5\%}$ (°C)	$T_{10\%}$ (°C)	$T_{50\%}$ (°C)	T_{peak} (°C)	Residue at T_{peak} (%)	Residue at 800°C (%)
			N_2			
**EPO**	295	347	381	370	65.6	8.42
**EPO_2%Si_2.32**	213	288	384	363	72.9	9.61
**EPO_6%Si_2.32**	239	300	404	377	66.6	16.7
			Air			
**EPO**	280	352	358	365	84.6	0.75
**EPO_2%Si_2.32**	231	300	398	370	73.3	3.37
**EPO_6%Si_2.32**	185	243	407	369	71.2	3.46

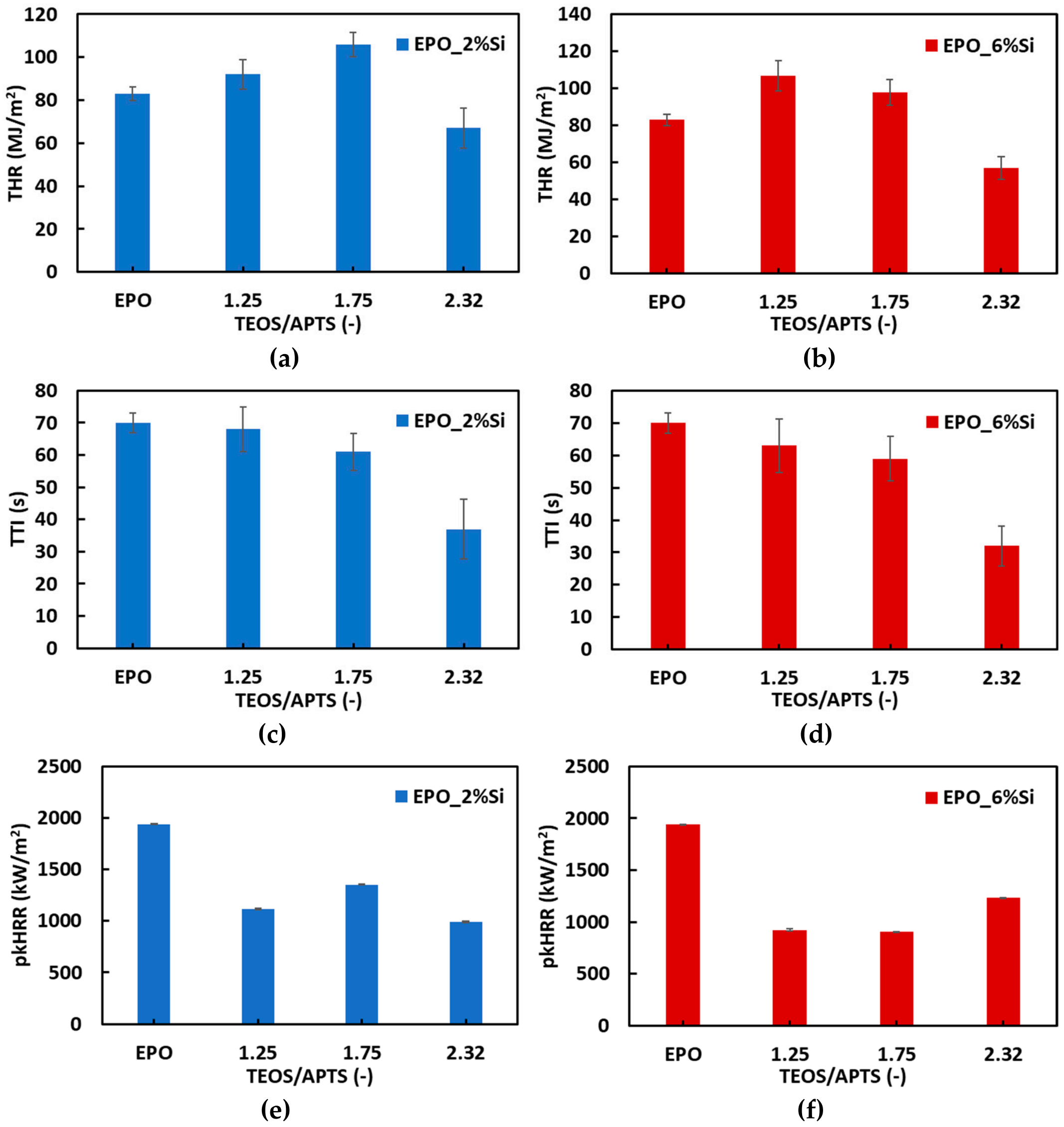

Figure S3. THR vs. TEOS/APTES molar ratio for the two investigated series containing (a) 2 wt.% of silica and (b) 6 wt.% of silica. TTI vs. TEOS/APTES molar ratio for the two investigated series containing (c) 2 wt.% of silica and (d) 6 wt.% of silica. pkHRR vs. TEOS/APTES molar ratio for the two investigated series containing (e) 2 wt.% of silica and (f) 6 wt.% of silica.
